# Effect of Beverages on the Surface Roughness and Staining of Modified Polyetheretherketone (PEEK) Materials

**DOI:** 10.3390/polym18121548

**Published:** 2026-06-22

**Authors:** Aybike Cengiz Dağtekin, Samet Tekin

**Affiliations:** Department of Prosthodontics, Faculty of Dentistry, Firat University, 23119 Elazig, Türkiye

**Keywords:** color stability, CAD-CAM dentistry, BioHPP, in vitro aging, surface topography

## Abstract

This study investigates color and surface alterations in neat and modified PEEK materials (10 × 3 mm, *n* = 140) immersed in various beverage solutions. Surface roughness (Ra) and color change (ΔE) were analyzed, supported by SEM and AFM evaluations. Specimens polished with 400–1200 grit sandpaper were measured via profilometry before a 30-day immersion in distilled water, coffee, tea, cola, and red wine (refreshed every 12 h). Post-immersion results indicated that material and solution types significantly influenced Ra and ΔE values (*p* < 0.05), with the TP group being the least affected. Statistically, tea caused the greatest discoloration. The GFP group exhibited the highest Ra, indicating inferior surface stability, whereas TP and CR groups remained below the 0.2 μm clinical threshold. Specifically, the TP group demonstrated the lowest Ra value. Although perceptible color changes occurred in all materials, a positive correlation was identified between material type and beverage solutions. SEM and AFM analyses confirmed the presence of surface micro-cracks and morphological irregularities.

## 1. Introduction

Advances in computer-aided design and manufacturing (CAD-CAM) technology have facilitated the development of new materials that can be machined with high precision for the fabrication of dental prostheses. Currently, polyetheretherketone (PEEK), a sub-member of the polyaryletherketone (PAEK) family, has emerged as a prominent material recommended for use in implants, temporary abutments, removable partial dentures (RPD), fixed prostheses, implant healing caps, and implant-supported hybrid prostheses. PEEK is a methacrylate-free, high-performance thermoplastic; it is a biomaterial that exhibits properties suitable for the dental and medical fields and is increasingly being preferred. The lack of branching in these highly aromatic polymers reflects the characteristic order of PAEK structures and contributes to their superior resistance to chemicals, radiation, heat, and other physical properties required of high-performance polymers. Despite being classified as rigid polymers, they exhibit a certain degree of flexibility owing to the alternating ketone and ether bonds within their structure [1]. PEEK is a material distinguished by its high biocompatibility, suitable mechanical properties, high-temperature resistance, chemical stability, polishing potential, low specific gravity, wear resistance, low plaque affinity, and strong bonding characteristics to composites and cements. When compared with alternative materials such as zirconia, glass ceramics, and metal alloys, its low modulus of elasticity—similar to bone—provides a biomechanical advantage [2]. Due to this property, it reduces the forces transmitted to the abutment teeth by absorbing functional stresses and potentially destructive forces [3].

Color stability is a critical feature for dental restorative materials. Any deviation from the standard color value indicates aging or structural damage of the material. Color changes observed in restorative materials during clinical use are generally caused by factors such as stain accumulation, dissolution of components, water absorption, alteration in optical properties, and surface roughness [4,5]. The threshold value for the color change value ΔE is accepted as 3.3 [6,7]. Rough surfaces exhibit a higher potential for stain retention compared to smooth surfaces, a condition that may adversely affect patient comfort. The accepted average surface roughness value (Ra) for dental restorations has been established as 0.2 µm [2,4]. Roughness measured with the aid of instruments is characterized by images possessing various heights, depths, and distances [8]. In the investigation of surface roughness, quantitative methods such as profilometry and qualitative methods such as atomic force microscopy (AFM) and scanning electron microscopy (SEM) are widely utilized [9]. AFM is widely utilized in the field of dentistry. It facilitates the acquisition of high-resolution three-dimensional images by scanning the topography of the contact surfaces of various materials at micro and nano levels. Consequently, a detailed analysis of material surfaces becomes possible [10]. SEM produces images by focusing an electron beam onto the surface of a specimen. The atoms and electrons in the specimen interact with each other to produce various signals. A three-dimensional image of the surface is obtained [11].

In contemporary prosthodontics, selecting the ideal framework or restorative material requires a careful balance between biological compatibility, mechanical durability, and optical longevity. Historically, polymethylmethacrylate (PMMA) based resins, dental composites, and monolithic zirconia have been utilized as gold standards for various fixed and removable prostheses [12,13,14,15]. However, traditional PMMA resins are highly prone to water sorption, intraoral structural fatigue, and subsequent discoloration over time, which often leads to clinical fractures under masticatory loads [14]. Monolithic zirconia provides absolute wear resistance and excellent color stability, yet its exceptionally high elastic modulus often triggers the stress shielding phenomenon, causing localized bone resorption around abutments or implants, alongside provoking three times more abrasive wear against natural antagonist teeth [13]. While indirect hybrid composites offer improved esthetics, their heterogeneous filler-matrix interfaces remain susceptible to localized hydrolytic surface alterations and severe beverage-induced extrinsic staining. Industrial CAD/CAM five-axis milling configurations and prefabricated PEEK disks possess minimized residual monomers and reduced structural porosity. This molecular density provides excellent chemical inertness and superior hydrophobicity, allowing native or structurally optimized PEEK variants to exhibit significantly lower staining susceptibility and lower baseline surface roughness compared to traditional PMMA and hybrid composites [12]. Consequently, understanding the long-term physical and optical behavior of modified PEEK matrices under distinct dietary challenges is critical to fully establishing their clinical lifespan against conventional restorative materials.

Although several studies have evaluated the discoloration and optical characteristics of neat PEEK and ceramic-filled PEEK (BioHPP), there is a critical gap in the dental literature regarding the long-term surface topography, roughness stability, and color resistance of specific innovative variants, such as titanium dioxide-filled PEEK and glass fiber-reinforced PEEK, when subjected to prolonged chemical and thermal exposure from frequently consumed dietary beverages. Investigating how these distinct inorganic fillers alter the material’s surface dynamics is essential for determining their longevity in clinical applications. Therefore, the novelty of this study lies in its comprehensive, comparative evaluation of both neat and structurally modified PEEK materials using a combined approach of quantitative profilometry and qualitative, high-resolution three-dimensional micro/nanometric analyses (SEM and AFM) following an extended aging protocol.

The objective of this study is to provide insights regarding the selection of suitable materials in terms of esthetics, color, and surface properties for neat PEEK and modified PEEK varieties, whose utilization is increasing alongside advancing technology. The primary research hypothesis established for the study is that beverage solutions will cause color changes in the materials. The secondary research hypothesis is that beverage solutions will lead to significant changes in the surface properties of the materials.

## 2. Materials and Methods

This study was approved by the Firat University Non-Interventional Ethics Committee (FUGOEK) (No. 2024-13-46).

### 2.1. Materials and Specimen Preparation

The sample size and distribution of the study groups were determined using G*Power analysis. Based on the power analysis, to test statistical significance at 80% power (power: 0.80) and a 5% significance level (α = 0.05), the minimum sample size for each group was determined to be *n* = 7, assuming an effect size d of 0.416 and a standard deviation of 1.64 for change in roughness. Sample size calculations were performed using the G*Power 3.0.10 (Franz Faul, University of Kiel, Kiel, Germany) software package.

Four distinct types of PEEK materials were evaluated in this study: neat PEEK, TiO_2_-filled PEEK, nanoceramic-filled PEEK, and glass fiber-reinforced PEEK. The neat PEEK, TiO_2_-filled PEEK, and nanoceramic-filled PEEK materials were commercially obtained in standard CAD-CAM disk formats (98 mm in diameter), and cylindrical specimens with dimensions of 10 × 3 mm were fabricated using an MCX5 milling machine (Dentsply Sirona, Bensheim, Germany). However, due to manufacturer supply limitations, the glass fiber-reinforced material (TECAPEEK GF30) was exclusively available in industrial cylindrical rod forms. To overcome this dimensional variance and obtain identical geometry, the glass fiber-reinforced PEEK rods were sectioned into 3 mm thick specimens using a precision cutting device (Struers Minitom; Struers, Copenhagen, Denmark). A total of 140 specimens were prepared, with 35 specimens allocated to each PEEK group.

To strictly eliminate any potential surface topography bias or systematic errors introduced by the different initial fabrication mechanisms (CAD-CAM milling versus precision cutting), a highly standardized surface preparation protocol was equally applied to all 140 specimens. The surfaces were first ground under water cooling using sequential 400, 800, and 1200 grit silicon carbide papers on a disk grinding device operated at 300 rpm for 5 s per specimen. To achieve a uniformly smooth surface, the specimens were then polished with a universal polishing paste (Ivoclar Vivadent AG, Schaan, Liechtenstein) using a tabletop micromotor (Strong 210, Saeshin, Daegu, Republic of Korea) at 1500 rpm for 90 s. Following the polishing phase, all specimens were cleaned in deionized water using an ultrasonic device for 30 s, immersed in distilled water for 10 min, and subsequently air-dried. After completing these pre-treatment procedures, seven specimens were randomly selected from each of the four material groups to be assigned to the five beverage setups, establishing twenty distinct experimental groups (Table 1).

### 2.2. Conducting Analyses

Following the pre-treatment procedures, the initial surface roughness value (Ra0) of all specimens was measured using a profilometer (Mitutoyo-SJ 410, Mitutoyo Corporation, Kawasaki, Japan). The profilometer resolution was set to 0.01 µm, with a cut-off length of 0.8 mm, a transverse length of 5.5 mm, and a probe tip speed of 1 mm/s. Each specimen was measured from three different regions, and the data were recorded. Color measurements of all specimens were conducted from three different points using a spectrophotometer (VITA Easyshade V, Vita Zahnfabrik, Bad Säckingen, Germany), which was calibrated at each repetition; the probe tip was positioned at a 90-degree angle to the specimen surface to standardize the evaluation position and exclude ambient light. All measurements were performed from a standardized distance under D65 illuminant conditions against a non-reflective gray background. The numerical L*a*b* values were recorded by calculating the average of the measurements. For surface characterization, one randomly selected specimen from each group was coated with gold–palladium to achieve a conductive surface and subsequently examined using scanning electron microscopy (SEM; Zeiss EVO MA10, Carl Zeiss AG, Oberkochen, Germany) at ×1000 magnification. The resulting images were recorded. One randomly selected specimen was analyzed via AFM (PARK XE7, Park Systems, Gyeonggi, Republic of Korea) using a 25 μm scan area, and 3D images were captured. Also, FTIR analysis was applied to investigate the chemical bonding structures and to evaluate potential degradation after the 30-day immersion period. The analyses were performed using a Nicolet Apex FTIR Spectrometer (Thermo Fisher Scientific, Waltham, MA, USA) via an ATR accessory in the range of 4000–650 cm^−1^ and with a resolution of 4 cm^−1^.

### 2.3. Solution Preparation

The specimens were immersed in various beverage solutions (distilled water, coffee, tea, cola, and red wine) for a period of 30 days. Solutions were refreshed twice daily at 12 h intervals and prepared fresh for each replacement. The 30-day immersion period and the 12 h solution replenishment interval utilized in this study were adopted from established dental literature protocols evaluating high-performance polymers. To maintain a constant concentration of active staining pigments, stabilize pH levels, and eliminate colorant sedimentation or microbial contamination over the prolonged aging framework, all immersion media were fully refreshed every 12 h [16]. The beverage solutions were prepared as follows: 200 mL of distilled water; for the coffee solution, 1 spoonful (2 g) of instant coffee was mixed with 200 mL of boiling water and steeped for 10 min; for the tea solution, one black tea bag was steeped in 200 mL of boiling water for 10 min; 200 mL of red wine; and 200 mL of cola. The staining solutions were prepared following a standardized protocol adapted from contemporary dental polymer literature [17]. Each specimen was individually numbered and stored in sealed plastic containers. Throughout the experiment, the specimens were maintained in a dark environment to simulate the intraoral atmosphere.

### 2.4. Repetition of Analyses

At the conclusion of the 30-day period, all specimens were removed from the beverage solutions, rinsed under running water for 4 min, and dried. Final surface roughness (Ra1) measurements of the 140 specimens were conducted using a profilometer under the same conditions as the baseline measurements, and the data were recorded. SEM and AFM analyses were performed on one randomly selected specimen from each group (totaling 20 specimens), and the images were captured. Color measurements were obtained from three different points on each of the 140 specimens using a spectrophotometer under identical conditions. The spectrophotometer was calibrated before each session. The averages of the recorded numerical L*a*b* values were calculated. ΔE values were determined based on the initial and final measurements according to the following equation:

(1) $$\Delta E^{*}=\sqrt{{\left(L2-L1\right)}^{2}+{\left(a2-a1\right)}^{2}+{\left(b2-b1\right)}^{2}}$$

### 2.5. Statistical Analysis

Statistical analyses of color changes were performed using GraphPad Prism 10.6.1 (GraphPad Software, LLC, Boston, MA, USA) software. The conformity of data to a normal distribution was evaluated with the Shapiro–Wilk test. According to the results of the test, the homogeneity of variances was evaluated using Levene’s test. The data showed a normal distribution (*p* > 0.05). The main effects and interactions of materials and beverages were evaluated with the two-way ANOVA test using the SPSS software package (IBM SPSS version 22.0) via the general linear model procedure. Additionally, two-way ANOVA was applied to examine the discoloration and roughness changes in each material within different beverages. To determine the groups with statistically significant differences, the Tukey HSD (Honest Significant Difference) post hoc test was utilized. The relationship between Ra0 and Ra1 was examined with the dependent *t*-test. Additionally, the systemic relationship between the magnitude of ΔE and the percentage change in Ra across the experimental cohorts was quantitatively evaluated using Pearson’s linear correlation analysis. In all analyses, a value of *p* < 0.05 was considered statistically significant.

## 3. Results

- As a result of the study, binary interactions between the type of solution and discoloration were found to be significant (*p* < 0.05). This condition indicates that the same material demonstrates different amounts of discoloration within different solutions.
- The interaction between material type and discoloration was found to be significant (*p* < 0.05). This result showed that the same solution causes different discoloration effects on different materials.
- Triple interactions between material type, beverage solution type, and discoloration were found to be significant (*p* < 0.05). When all parameters were combined, the materials exhibited distinct discoloration characteristics.

### 3.1. Surface Roughness

#### 3.1.1. The Change in Roughness of the Same Material Within Different Solutions

**Figure 1:**
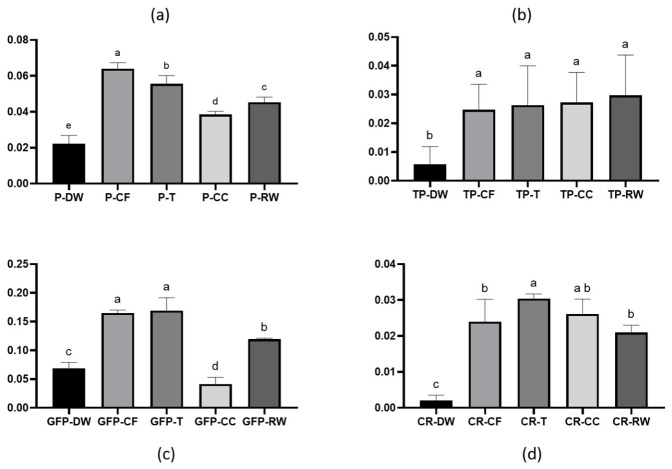
Graphs of ΔRa changes for materials in various solutions. (**a**) ΔRa change graph of pure PEEK in different solutions; (**b**) ΔRa change graph of titanium-filled PEEK in different solutions; (**c**) ΔRa change graph of glass fiber-filled PEEK in different solutions; (**d**) ΔRa change graph of nanoceramic-filled PEEK in different solutions. a–e: There is a statistically significant difference between materials bearing different lowercase letters (*p* < 0.05).

Material P was affected by the solutions to varying degrees. An increase in roughness occurred in the following order from most to least: coffee, tea, red wine, cola, and distilled water. The roughness change in TP group specimens did not show a statistically significant difference in tea, coffee, cola, and wine solutions (*p* > 0.05). Distilled water exhibited the lowest roughening effect (*p* < 0.05). In the TP group, the average Ra1 values measured at the end of 30 days were below the threshold value of 0.2 μm in all groups. In the GFP group, coffee and tea caused the highest increase in roughness, and there is no statistically significant difference between them (*p* > 0.05). Among the GFP groups, the solution that caused the lowest surface roughness was cola. In the GFP group, the average Ra1 values measured at the end of 30 days were above the threshold value of 0.2 μm in all groups. In the CR material, the solution that caused the most roughness change was tea; however, the CR-T and CR-CC groups did not show a statistically significant difference (*p* > 0.05). Similarly, no significant difference was observed between CR-CC and CR-CF (*p* > 0.05). In the CR group, the average Ra1 values measured at the end of 30 days were below the threshold value of 0.2 μm in all groups (Figure 1).

#### 3.1.2. The Change in Roughness Caused by the Same Solution on Different Materials

**Figure 2:**
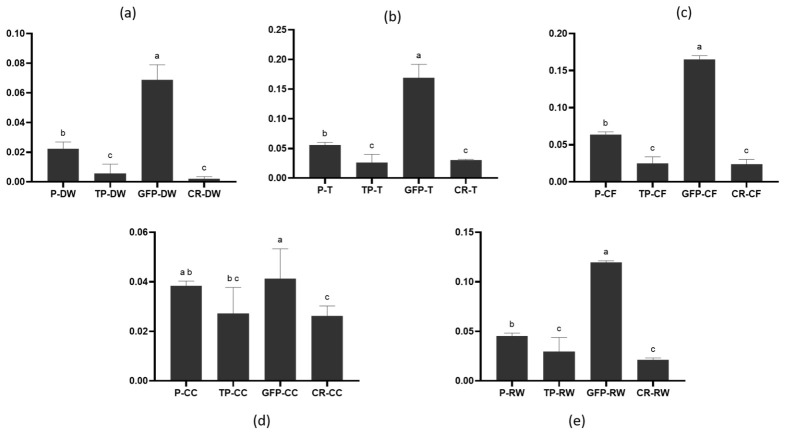
Graphs of ΔRa changes across different materials within the same solution. (**a**) ΔRa change graph of distilled water in different materials; (**b**) ΔRa change graph of tea in different materials; (**c**) ΔRa change graph of coffee in different materials; (**d**) ΔRa change graph of cola in different materials; (**e**) ΔRa change graph of red wine in different materials. a–c: There is a statistically significant difference between materials bearing different lowercase letters (*p* < 0.05).

When examining the effect of distilled water on different materials, the highest roughness change occurred in the GFP group, showing significant differences from all other groups (*p* < 0.05). The TP and CR groups were the least affected by distilled water in terms of roughness change, with no statistically significant difference between them (*p* > 0.05). The P group was moderately affected by distilled water. The group whose surface properties changed the most due to the influence of coffee was once again the GFP group, as was the case with all other beverages. The second most affected group was the P group. The TP and CR groups showed no statistically significant difference (*p* > 0.05) and were the groups least affected by coffee. While the Ra0 value of the GFP group was 0.167 ± 0.010 μm, the Ra1 value measured after immersion in the solution increased to 0.332 μm. The roughness value increased approximately twofold. In the tea solution, the GFP group exhibited highly significant statistical differences compared to the other groups (*p* < 0.05) and was the group with the highest roughness change. Similar to its behavior in coffee, the surface roughness of the GFP material increased approximately twofold in tea. The second most affected group by tea was the P group. No statistically significant difference was observed between the TP and CR groups (*p* = 0.9379). TP and CR group materials were the groups least affected by tea. Among the groups immersed in cola, the GFP group exhibited the highest degree of surface roughening. However, it did not show a statistically significant difference compared to the P group (*p* = 0.9159). No statistically significant difference was observed between the P and TP groups either (*p* = 0.0822). Furthermore, the TP and CR groups exhibited similar surface changes in cola (*p* = 0.9938). When examining the change in roughness within red wine, the group most affected was again the GFP group. The GFP group exhibited highly significant differences from all other groups (*p* < 0.05). Following the GFP group, the group most affected by red wine was the P group. Among the groups, the groups least affected by red wine were the CR and TP groups, and they did not show a statistically significant difference (*p* > 0.05) (Figure 2).

The lowest initial Ra0 values were observed in the TP group, exhibiting statistically significant differences compared to the other groups. The highest Ra0 values were recorded in the GFP and P groups, with no statistically significant difference between them (*p* > 0.05). The CR group displayed Ra0 values intermediate to those of the other groups. When comparing Ra1 values across all solutions for different materials, the TP group again showed the lowest roughness, while the GFP group exhibited the highest values. Both the TP and CR groups demonstrated Ra1 values below the threshold (Table 2).

### 3.2. Color Stability

#### 3.2.1. The Change in Color of the Same Material Within Different Solutions

**Figure 3:**
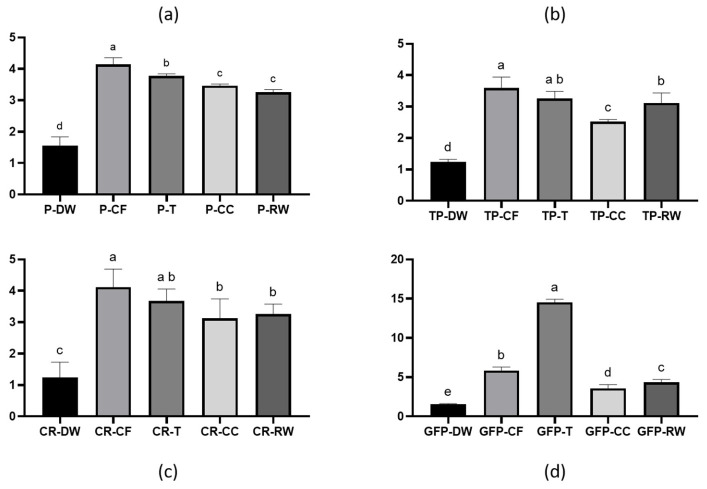
Graphs of ΔE changes for materials in various solutions. (**a**) ΔE change graph of pure PEEK in different solutions; (**b**) ΔE change graph of titanium-filled PEEK in different solutions; (**c**) ΔE change graph of glass fiber-filled PEEK in different solutions; (**d**) ΔE change graph of nanoceramic-filled PEEK in different solutions. a–e: There is a statistically significant difference between materials bearing different lowercase letters (*p* < 0.05).

It was observed that pure PEEK materials exhibited the highest degree of discoloration in coffee. Following coffee, tea was statistically determined to be the solution causing the most significant discoloration. While cola and red wine caused similar color changes in the pure PEEK groups (*p* > 0.05), distilled water resulted in the least amount of color change. The most significant color change among the TP groups occurred in coffee. No statistically significant difference was observed between tea and coffee solutions when compared against the TP groups (*p* = 0.0910). Apart from the control group (distilled water), cola was the beverage that caused the least discoloration. The GFP material exhibited the greatest discoloration in tea. Based on the mean ΔE values, the GFP-T group was observed to undergo approximately nine times more discoloration compared to the control group (GFP-DW). The solutions causing discoloration in the GFP material were, in descending order: tea, coffee, red wine, cola, and distilled water. In all solutions except distilled water, the ΔE values of the GFP groups exceeded the threshold value. Coffee and tea were the solutions that induced the highest level of discoloration in the CR group (*p* > 0.05). When the mean ΔE values for the TP group were analyzed, it was observed that coffee and tea solutions produced changes exceeding the clinical threshold. No statistically significant differences were found among the CR-T, CR-CC, and CR-RW groups (*p* > 0.05) (Figure 3).

#### 3.2.2. The Change in Color Caused by the Same Solution on Different Materials

**Figure 4:**
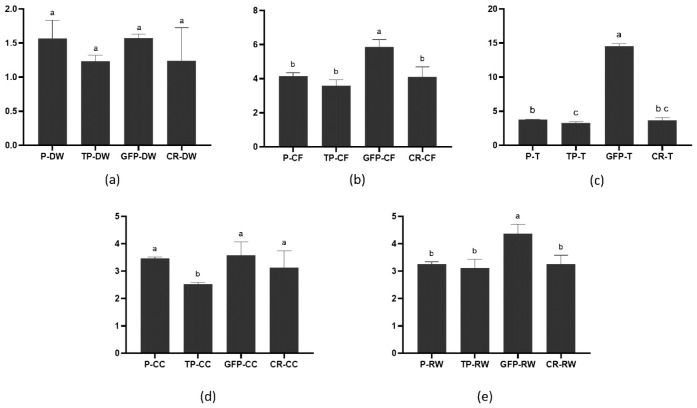
Graphs of ΔE changes across different materials within the same solution. (**a**) ΔE change graph of distilled water in different materials; (**b**) ΔE change graph of coffee in different materials; (**c**) ΔE change graph of tea in different materials; (**d**) ΔE change graph of cola in different materials; (**e**) ΔE change graph of red wine in different materials. a–c: There is a statistically significant difference between materials bearing different lowercase letters (*p* < 0.05).

No statistically significant differences in color change were observed among the groups immersed in distilled water (*p* > 0.05). All materials exhibited values below the threshold of 3.3 ΔE. In coffee, the highest ΔE values were identified in the GFP group. The GFP-CF group exhibited significantly higher discoloration values compared to other materials (*p* < 0.05). Excluding this group, no statistically significant differences were observed between the other groups (*p* > 0.05). Among the sample groups immersed in tea, the GFP group again exhibited the highest degree of color change. The TP group was the least discolored in the tea solution. The GFP group showed approximately 4.5 times more discoloration than the TP group in tea (*p* < 0.05). Statistical evaluation revealed significant differences between the P and TP groups (*p* < 0.05). The CR group served as a transitional group between the TP and P groups. Among the groups immersed in cola, the TP group exhibited the least discoloration and was found to be statistically significantly different from the other groups (*p* < 0.05). No statistically significant difference was detected among the remaining groups within the cola solution (*p* > 0.05). Considering the mean ΔE values, the TP and CR groups were the only materials immersed in cola that did not exceed the ΔE threshold value. The GFP group, which was the only group to exceed the ΔE threshold in red wine, exhibited a statistically significant difference compared to the other groups (*p* < 0.05). No significant differences were observed among the remaining groups (*p* > 0.05) (Figure 4).

The GFP group exhibited the highest degree of discoloration among all materials. The P and CR groups showed the second highest levels of discoloration, with no statistically significant difference observed between them (*p* > 0.05). Among all materials, the TP group was the most resistant to beverage solutions, displaying the lowest discoloration values and showing statistically significant differences compared to the other groups (*p* < 0.05). The TP group exhibited changes below the critical ΔE value in nearly all samples. It was also determined to be the most resistant group to both discoloration and surface roughness changes. Statistical analysis of the discoloration levels induced by all beverage solutions revealed significant differences among all beverages. The solutions causing discoloration, in descending order, were tea, coffee, red wine, cola, and distilled water.

### 3.3. Correlation Analysis Between Color Stability and Surface Roughness

Pearson’s linear correlation analysis demonstrated a statistically significant, strong positive correlation between ΔE and Ra (r = 0.7332, 95% confidence interval: 0.4303 to 0.8877, *p* = 0.0002). The calculated coefficient of determination (r^2^ = 0.5376) mathematically confirmed that approximately 53.76% of the total variance in chromatic shift across the evaluated PEEK variants can be directly accounted for by the surface roughness acceleration.

### 3.4. Surface Morphology (SEM)

**Figure 5:**
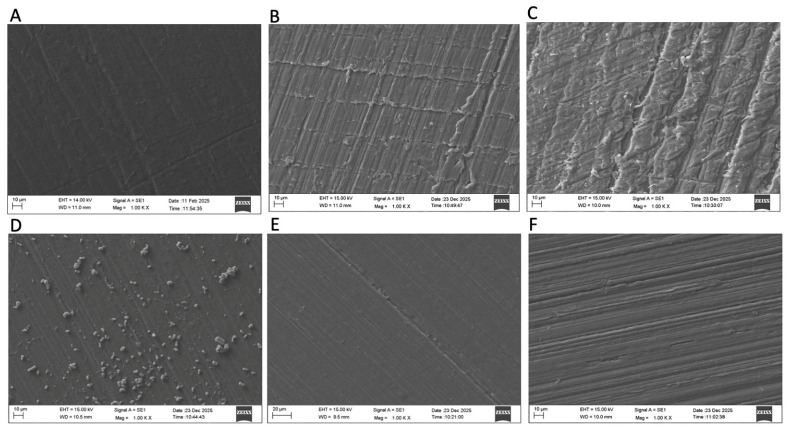
Initial SEM image of the P group (**A**), SEM image of the P-DW group (**B**), SEM image of the P-CF group (**C**), SEM image of the P-T group (**D**), SEM image of the P-CC group (**E**), and SEM image of the P-RW group (**F**).

**Figure 6:**
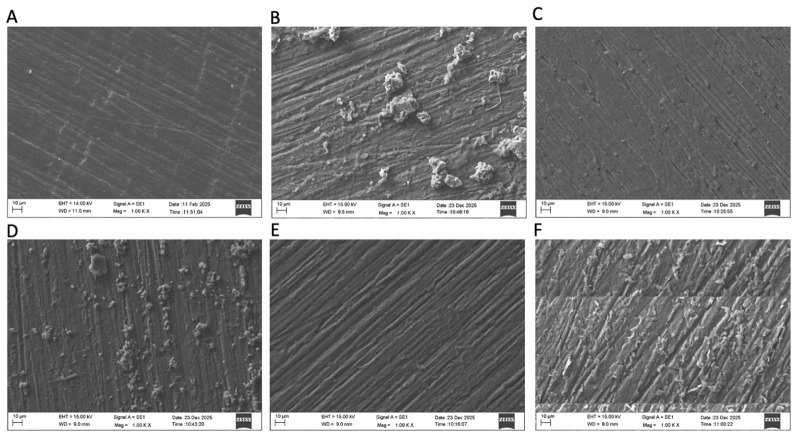
Initial SEM image of the TP group (**A**), SEM image of the TP-DW group (**B**), SEM image of the TP-CF group (**C**), SEM image of the TP-T group (**D**), SEM image of the TP-CC group (**E**), and SEM image of the TP-RW group (**F**).

**Figure 7:**
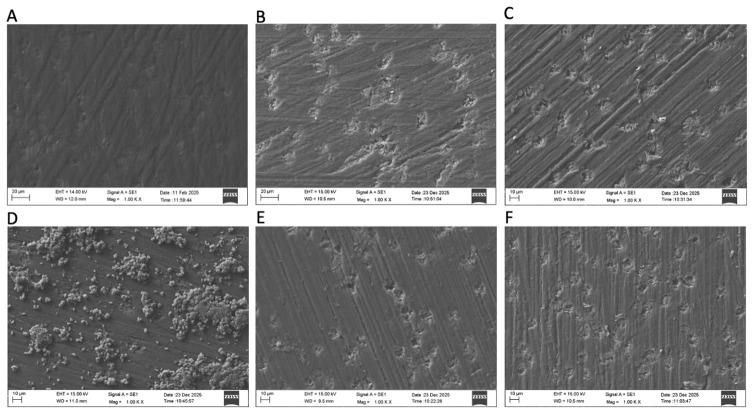
Initial SEM image of the GFP group (**A**), SEM image of the GFP-DW group (**B**), SEM image of the GFP-CF group (**C**), SEM image of the GFP-T group (**D**), SEM image of the GFP-CC group (**E**), and SEM image of the GFP-RW group (**F**).

**Figure 8:**
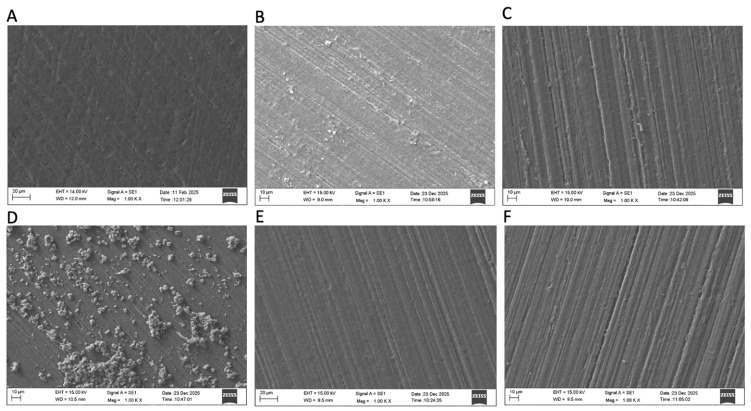
Initial SEM image of the CR group (**A**), SEM image of the CR-DW group (**B**), SEM image of the CR-CF group (**C**), SEM image of the CR-T group (**D**), SEM image of the CR-CC group (**E**), and SEM image of the CR-RW group (**F**).

The homogeneous surface characteristics of the materials prior to thermal aging were replaced by distinct morphological changes following the aging process. Surface irregularities and topographic variations observed during this process exhibited material-specific differences through comparative analyses of SEM images. Notably, images for GFP-PEEK revealed the presence of spherical void structures even at baseline, which increased upon immersion in beverages. Generally, samples of all materials immersed in black tea exhibited extensive surface deposits. While groups immersed in cola showed less surface roughness alterations, a predominant appearance of pronounced topographical valleys was observed in coffee (Figure 5, Figure 6, Figure 7 and Figure 8).

### 3.5. Microscopic Analysis (AFM)

**Figure 9:**
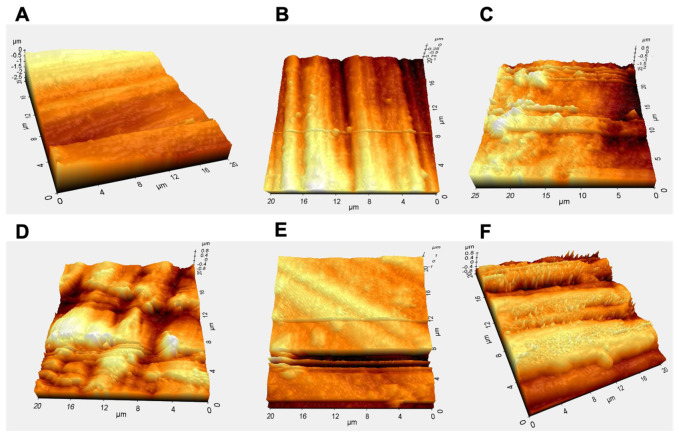
Initial AFM image of the P group (**A**), AFM image of the P-DW group (**B**), AFM image of the P-CF group (**C**), AFM image of the P-T group (**D**), AFM image of the P-CC group (**E**), and AFM image of the P-RW group (**F**).

**Figure 10:**
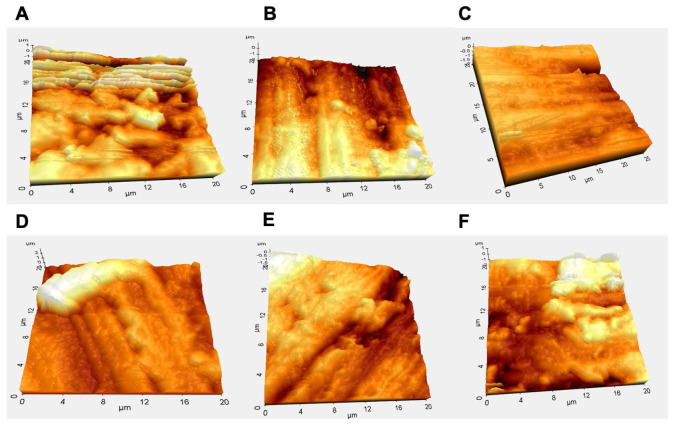
Initial AFM image of the TP group (**A**), AFM image of the TP-DW group (**B**), AFM image of the TP-CF group (**C**), AFM image of the TP-T group (**D**), AFM image of the TP-CC group (**E**), and AFM image of the TP-RW group (**F**).

**Figure 11:**
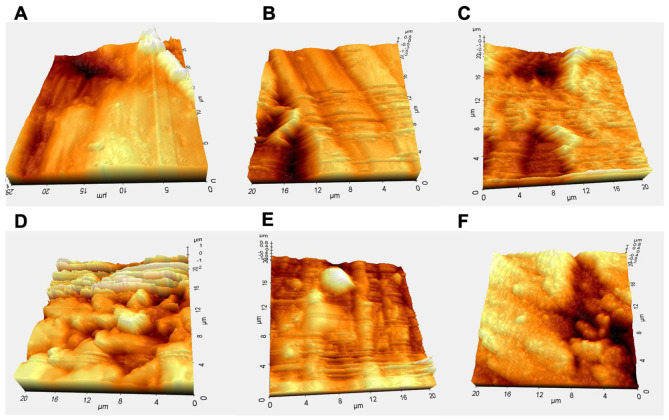
Initial AFM image of the GFP group (**A**), AFM image of the GFP-DW group (**B**), AFM image of the GFP-CF group (**C**), AFM image of the GFP-T group (**D**), AFM image of the GFP-CC group (**E**), and AFM image of the GFP-RW group (**F**).

**Figure 12:**
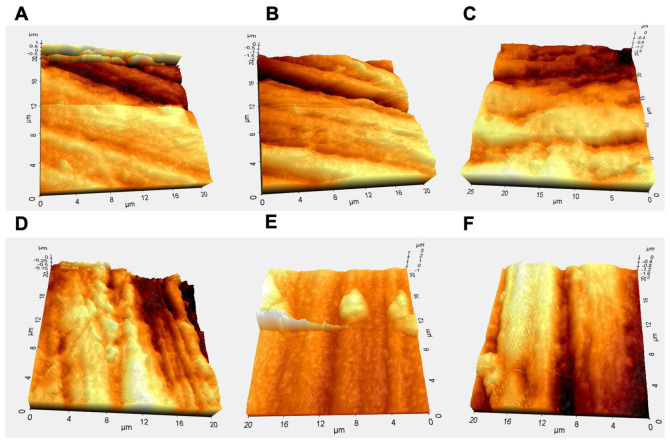
Initial AFM image of the CR group (**A**), AFM image of the CR-DW group (**B**), AFM image of the CR-CF group (**C**), AFM image of the CR-T group (**D**), AFM image of the CR-CC group (**E**), and AFM image of the CR-RW group (**F**).

In the AFM analysis of the materials, minimal surface roughness alterations were observed with distilled water and cola solutions, whereas topographical irregularities were quite substantial in materials immersed in black tea and coffee. Extensive peaks and valleys were observed in coffee and tea. The cross-sectional amplitude values in coffee and tea were found to be higher compared to the other solutions (Figure 9, Figure 10, Figure 11 and Figure 12).

### 3.6. FTIR Spectroscopy Analysis

**Figure 13:**
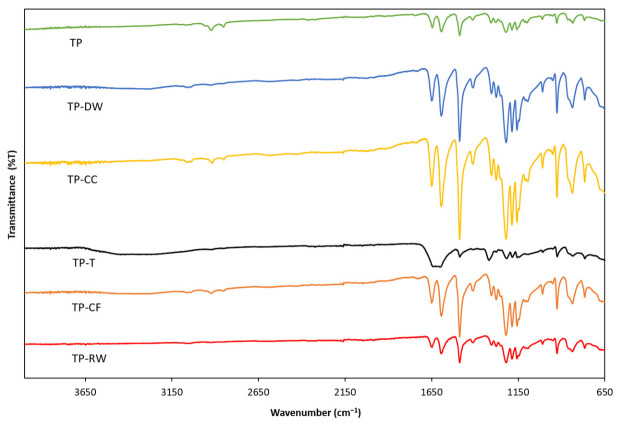
FTIR spectra of the TP group after 30 days of interaction with different solutions (distilled water, cola, tea, coffee and red wine).

Comparative FTIR spectra of the pristine and beverage-modified PEEK surfaces after 30 days of immersion are shown in Figure 13. To maintain conciseness, the titanium-filled PEEK (TP) group was selected as a representative baseline since all modified PEEK variants displayed identical structural footprints and parallel chemical degradation/adsorption trends.

The pristine matrix exhibited characteristic PEEK peaks: carbonyl (C=O) stretching at 1647 cm^−1^, aromatic ring (C=C) vibrations at 1597 cm^−1^ and 1487 cm^−1^, and asymmetric diaryl ether (C-O-C) linkages between 1218 cm^−1^ and 1150 cm^−1^. Following 30 days of aging, solution-specific surface alterations were observed:

- Distilled Water (DW): Retained nearly identical peak configurations with the pristine control, verifying the outstanding hydrolytic stability and fluid-sorption resistance of the polymer matrix.
- Cola (CC): Acidic immersion led to a slight sharpening and increase in the intensities of carbonyl and ether bands, suggesting a micro-etching effect on the outer amorphous layers. Emerged aliphatic C-H vibrations at 2847 cm^−1^ and 2915 cm^−1^ indicated trace organic residues.
- Tea (T): Exhibited the most dramatic modification; a broad, intense hydrogen-bonded hydroxyl (O-H) band appeared at approximately 3300 cm^−1^, along with new aliphatic C-C skeletal peaks at 1136 cm^−1^, 1185 cm^−1^, and 1316 cm^−1^. These findings demonstrate that tea polyphenols and tannic acids adsorbed onto the surface, forming a dense organic layer that physically masked the underlying matrix.
- Coffee (CF): Introduced secondary peaks associated with adsorbed coffee components, including caffeine (C-N stretching at 1407 cm^−1^), chlorogenic acid (C-OH deformation at 1304 cm^−1^ and ester linkages at 1275 cm^−1^), and polysaccharides (C-O bands at 1093 cm^−1^). The core PEEK peaks remained unaffected, showing that these components merely formed a superficial film.
- Red Wine (RW): Caused a prominent reduction in the intensities of all primary polymer peaks (1650 cm^−1^, 1595 cm^−1^, and 1220 cm^−1^). This indicates a physical plasticization effect on the surface chains induced by the synergistic combination of ethanol and organic acids within the wine.

## 4. Discussion

PEEK materials are currently utilized in numerous applications, including implants, abutments, and removable and fixed prosthetic restorations [18,19,20]. Due to its superior mechanical properties, low water sorption, biocompatibility, and non-metallic esthetic structure, PEEK is considered a strong alternative to metal alloys in contemporary dentistry [21]. In addition to their biomechanical advantages, the PEEK-based materials selected for this study were intended to be evaluated in terms of surface stability. Pure PEEK material may exhibit limitations in certain clinical applications due to its bioinert nature and low surface energy. To address these shortcomings, Ahmad et al. reported that the mechanical properties and bioactivity of the material could be enhanced by incorporating titanium dioxide, ceramic particles, or carbon/glass fibers into the PEEK matrix [22]. The investigation of PEEK groups with various modifications in our study aims to reveal the effects of these modifications on the material’s surface stability and color resistance. Although it has been reported in the literature that the chemical structure of PEEK material exhibits high resistance to acidic and chromatic agents, data regarding how inorganic fillers incorporated into the material influence this resistance remain limited [3,12,23,24]. However, the chemical composition and hydrophilic character of materials directly affect fluid absorption and the subsequent stability loss within the oral environment. Therefore, evaluating the performance of developed modified PEEK types against the chemical and physical stresses induced by frequently consumed beverages is of critical importance for clinical material selection. Surface roughness is a decisive parameter for the long-term success of restorative materials in terms of plaque retention, staining, and wear resistance [25]. Surface roughness accelerates the wear of restorative materials, leads to discoloration, and increases plaque formation [26,27]. In the literature, the critical Ra value for bacterial adhesion and plaque accumulation is accepted as 0.2 μm [28]. Restorative materials must maintain their long-term durability within the oral environment. Considering intraoral conditions, materials are subjected to the continuous influence of both organic and inorganic components and salivary fluid. Consequently, chemical agents present in consumed food and beverages, along with variations in pH and temperature, play a decisive role in the longevity of prosthetic restorations [29]. Beverages with low pH levels accelerate the surface alteration process by affecting not only natural tooth surfaces but also the surfaces of other restorative materials. Studies have demonstrated that these materials undergo structural surface modifications when exposed to acidic environments [30]. To analyze the surface topography and roughness of restorative materials, various instruments such as mechanical and optical profilometers, SEM, and AFM are generally utilized. Profilometers, in particular, are frequently preferred methods among clinicians and researchers due to their lack of sample preparation requirements and their capacity for reproducible measurements. Additionally, AFM provides a comprehensive evaluation of surface measurement by enabling high-resolution, three-dimensional (3D) imaging of the material surface at a nanometric resolution [7]. AFM, utilized in nanoparticle and nanomaterial characterization, provides qualitative and quantitative data for a broad range of surfaces, including polymers, ceramics, composites, glass, and biological materials. This analytical method is of critical importance for materials science applications, providing detailed information on various physical properties such as size, shape, surface texture, and roughness. The acquired images enable a comprehensive visual and quantitative evaluation of surface characteristics [31]. For this reason, profilometry, AFM, and SEM analyses were preferred in our study to visualize surface roughness and surface topography.

Studies involving beverage solutions have utilized staining agents such as coffee, tea, cola, and red wine [32]. Coffee is widely recognized as a major staining agent for dental restorations due to high daily consumption rates [33,34]. Our statistical analysis revealed a significant positive correlation between discoloration and increased roughness (*p* < 0.05). Rough surfaces compromise cleansability and provide larger areas for the mechanical retention of coffee pigments [35]. Consequently, the high-roughness GFP group exhibited the greatest color change and roughness increase, while the structurally optimized TP group showed the highest resistance to these changes.

The formulas employed in the analysis of color changes directly affect the reliability of the dental material evaluations. In this study, the widely validated and standardized traditional CIE L*a*b* formula was preferred to calculate the total color shifts, ensuring a direct and precise objective assessment of the optical data within the established limits of clinical acceptability (ΔE ≤ 3.3) [6].

To simulate the long-term optical behavior of the materials, a 30-day immersion protocol was implemented. Heshmat et al. [36] and Kaya et al. [37] stated that in a similar protocol, a 28-day immersion period is equivalent to approximately 2.5 years of use in clinical conditions. The data obtained in our study provide significant insights into the medium-to-long-term esthetic lifespan of the materials.

When examining the effects of the solutions on the degree of discoloration, minimal color changes were observed in the samples immersed in distilled water (control group), though these changes were not statistically significant (*p* > 0.05). Similarly, Darabi et al. [38] noted that distilled water could cause slight discoloration in materials, attributing this phenomenon to the leaching of soluble components from the material during water absorption. In a study involving pure PEEK, Porojan et al. reported that distilled water caused slight discoloration by reducing microhardness; however, these changes were not clinically perceptible and were not found to be statistically significant [2]. In our study, we believe that the highest water absorption occurred in the GFP group due to the micro-cracks between the glass fibers, and therefore, the highest level of discoloration was observed in this group. In this study, tea and coffee induced the highest discoloration across all groups. Although some literature suggests coffee causes higher staining due to low-polarity pigment penetration [39,40] tea caused more aggressive discoloration in our findings, particularly for the GFP group. This can be attributed to distinct staining mechanisms: coffee pigments typically penetrate the polymer network via absorption, whereas tea stains primarily through surface adsorption [36,38]. The high surface roughness of the GFP group, combined with elevated beverage temperatures, accelerated the mechanical retention and adsorption of tannic acid from the tea solution [16]. Our SEM observations directly confirm this extensive surface deposit accumulation.

When evaluating the acidic beverages cola and red wine, it was found that cola induced a lower color change compared to coffee and tea, despite having a low pH value (approximately 2.5). This finding supports the view that surface erosion caused by acidic solutions tends to dissolve and remove the colored layer and reduce pigment retention [16,40]. Papathanasiou et al. [16] reported that cola caused less color change than coffee and wine, despite having the lowest pH value. Polychronakis et al. [41], on the other hand, suggested that the penetration of pigments in wine into the polymer surface remains limited despite their dense structure. In our study, the fact that the color change induced by cola and wine solutions remained near or below clinical threshold values supports the notion that acidic erosion might have a removal effect by dissolving the stained surface layer. The discoloration effect of the acidic red wine solution on the materials was more limited compared to coffee. Kaya et al. [37] proposed that the low ethanol content and dense structure of wine hinder pigment adhesion and penetration into the material surface, thus exhibiting lower ΔE values compared to coffee. Our findings also support this view, indicating that the color change induced by wine remains below or at the limit of clinical threshold values.

The color stability of PEEK material is derived from the inert properties of its chemical structure. Hamid et al. reported in their study that PEEK displays a more resistant profile against staining agents compared to other dental polymers such as polyamide and PMMA, but emphasized that discoloration may be unavoidable with long-term use, especially in aggressive solutions like coffee [21]. In our study, it was found that solutions with high staining capacity, such as tea and coffee, induced the greatest changes in color and roughness. Coffee was the solution that caused the highest color change in all groups except for the GFP group. The occurrences of fiber pull-out likely observed in the GFP group serve as evidence supporting the numerical data obtained.

When examining the effect of beverage type on materials, the findings for neat PEEK are in alignment with the study results of Mohamed and Eltamimi [42]. The researchers reported that, at the end of a 6-week immersion period, tea and coffee solutions caused statistically similar and significant levels of color change in PEEK material, whereas cola and distilled water produced minimal and similar effects on the material. This suggests that the PEEK surface exhibits resistance to hot and intensely pigmented solutions up to a certain saturation point, but the superficial accumulation of staining particles is inevitable during long-term exposure. Similarly, in our study, when considering all materials, coffee and tea caused average changes of 4.4 and 6.2 ΔE, respectively, while cola and red wine caused average changes of 3.1 and 3.4 ΔE. The fact that tea and coffee induced changes well above the threshold value is significant for the study. Joshua et al. [43] stated that the semi-crystalline molecular structure and low water absorption capacity of PEEK limit the penetration of staining agents into the matrix, thereby maintaining the optical integrity of the material. Due to the positive effects of PEEK, modified PEEK types that have been examined in a limited number of studies in the literature were preferred in our study. In particular, no similar study has been encountered in the literature investigating the changes in color and surface roughness of the GFP and TP groups.

When examining the material groups, the lowest color change and highest surface stability were observed in the TP group. This resistance can be attributed to the high opacity and refractive index provided to the material by titanium dioxide particles. Alkahtany et al. [44] reported that TiO_2_ addition masks color change by protecting the polymer matrix against ultraviolet light and external factors. Furthermore, it is thought that TiO_2_ reduces water absorption and limits surface alterations by strengthening the polymer structure. It has also been stated that TiO_2_ increases the hydrophobic property in the structure. Our findings also confirm these protective effects. Fonseca et al. [45] found that materials with increased hydrophobic properties experience less discoloration due to decreased water absorption. Therefore, we believe that the TP group in our study, which contains TiO_2_, exhibited less discoloration thanks to its high hydrophobicity. At the end of 30 days, the TP material exceeded the threshold value only in coffee, showing a 3.5 ΔE value. In all other solutions, it remained below the 3.3 threshold, demonstrating remarkably high physical stability. Additionally, it exhibited the lowest initial surface roughness among all materials, displaying very low initial Ra values such as 0.12 μm. When the Ra values measured at the end of 30 days are considered, in addition to showing the lowest increase in roughness, it also exhibited values below the threshold across all solutions, with an average of 0.16 μm. In contrast, the GFP group exhibited the highest color change and surface roughness values in all solutions, exceeding clinical thresholds in every solution except distilled water. The primary reason for this failure is the weak interface bonding between the inorganic glass fibers and the organic PEEK matrix. Tang et al. [46] stated that this weak bonding creates micro-channels for liquid penetration. More importantly, the separation of glass fibers from the matrix upon contact with liquid, creating micro-voids, allowed pigments to fill these gaps and led to irreversible discoloration. Furthermore, the fact that GFP samples in our study were prepared using a precision cutting device, unlike the other groups, is considered another factor that facilitated pigment retention by increasing initial surface roughness. It has been reported that the precision cutting process can create more surface defects initially compared to CAD-CAM milling [47]. In the study conducted by Fadhil et al., the color stability of PEEK materials produced via CAD-CAM and 3D printing was evaluated in coffee, tea, and cola. Consistent with our study, the solutions causing the highest color change were tea, coffee, and cola, respectively. Moreover, PEEK materials produced with CAD-CAM exhibited less color change due to their lower roughness compared to other materials [48]. This situation corroborates the reason for the high surface roughness of the GFP samples produced with a precision cutting device in our study.

When evaluating the relationship between color stability and surface morphology, Saraswathy et al. [35] identified a strong positive correlation between initial surface roughness and color change in PEEK material. Similarly, Papathanasiou et al. [16] emphasized that differences in hardness between inorganic filler particles and the matrix can create micro-irregularities on the surface after polishing, which increases stain retention. The high discoloration values observed in the GFP group in our study can be explained by the increase in surface roughness facilitating pigment accumulation, as suggested by these studies. Furthermore, the hydrophilic character and water absorption capacity of the materials are factors that directly affect color stability. Consistent with the findings of this study, the high surface roughness and discoloration observed in the GFP group can be explained by the hydrolytic damage caused by acidic and hot beverages at the fiber-matrix interface. The cracked structure observed in the AFM images of the GFP group may also have formed for this reason. The GFP group exhibited great changes, particularly in tea, reaching values such as 14.5 ΔE. This suggests that the use of glass fiber-reinforced PEEK in the intraoral region, especially without veneering, is not highly suitable.

PEEK and BioHPP groups exhibited moderate levels of color change. The stable performance of BioHPP compared to pure PEEK in terms of surface roughness increase can be explained by ceramic fillers reducing water absorption and lowering surface energy [49]. In our study, the P and CR groups showed no significant difference, exhibiting statistically similar ΔE values across all solutions. Furthermore, both groups were stained above the clinical limit in aggressive solutions such as coffee and tea. The esthetic performance of PEEK materials is closely related to their optical properties. Porojan et al., in their study investigating the translucency and opalescence parameters of BioHPP material, reported that the translucency values obtained were in the range of 1.25–3.60, which is quite low compared to natural tooth enamel and other esthetic restorative materials [3]. This finding highlights the limitations of the CR group materials in our study regarding the imitation of natural tooth appearance and underscores that their opaque structure is a factor limiting their use in the esthetic zone.

The relationship between color stability and surface roughness becomes even more complex with wear. In the study by Abhay et al., it was determined that the wear resistance of PEEK crowns is lower compared to zirconia. Wear in the oral environment increases surface roughness by creating micro-scratches and craters on the surfaces of materials [13]. When combined with our study’s finding that increased roughness accelerates discoloration, it becomes evident that the esthetic lifespan of PEEK composites may be shortened due to surface alterations under high wear loads. It was observed that the roughness values in the GFP and P groups exceeded the threshold limit and increased to a statistically significant level. The significant roughness increase after immersion can be attributed to the combined effects of chemical composition and temperature. Hot beverages like tea and coffee trigger micro-crack formation by creating a thermal cycling effect within the polymer matrix [16]. In the GFP group, this thermal and chemical stress caused severe hydrolytic interfacial separation due to the polarity difference between the PEEK matrix and the glass fillers [46]. This incompatibility led to fiber pull-out and matrix erosion, which is visually supported by the deep valleys and irregular peaks in our AFM images. Thus, hot beverages not only discolor the materials but also compromise their structural surface integrity [50]. Sevinç et al. reported that PEEK specimens maintained their mechanical properties after immersion in cola and even exhibited higher resistance values compared to other groups [51]. The researchers attributed this to the chemically inert nature of PEEK and the lack of absorption by the material due to the high polarity of cola. Our findings also support this chemical resistance mechanism, as acidic solutions such as cola had a limited effect on all groups. Statistical analysis revealed that, among the staining solutions (excluding distilled water), the lowest change in roughness occurred in the specimens immersed in cola. The reason for cola exhibiting less staining compared to other beverages may be associated with the tendency of the layers accumulated on the specimen surface to dissolve and return to the solution medium [39]. In our study, cola caused an average ΔE increase of 3.171 ± 0.561 across all materials, representing a change below the threshold value. Porojan et al. [23] observed that Ra values significantly decreased in PEEK specimens kept in acidic fruit juice, explaining this phenomenon by the acidic medium chemically dissolving surface irregularities to create a more homogeneous surface. In our study, the low roughness values observed in the cola groups can also be associated with a similar surface regulation mechanism.

Papathanasiou et al. [52] immersed BioHPP, PMMA, polyamide, and resin specimens in coffee, cola, red wine, and distilled water for 30 days and evaluated the changes in color and surface roughness. At the end of the study, the highest color change was observed in the coffee groups, while the lowest color change was observed in the cola groups. Additionally, PEEK containing nano-ceramics showed the least staining compared to other materials. In our current study, according to the statistical analysis of the BioHPP groups, the increase in color change was observed in coffee, tea, red wine, and cola solutions, respectively. High color stability is explained by nano-ceramic-reinforced PEEK being a semi-crystalline polymer and its advantageous chemical properties. These advantages include high temperature and chemical resistance, as well as low surface energy, minimal water solubility, and water absorption. These properties increase the resistance of BioHPP to surface alterations and significantly prevent the adhesion of extrinsic staining agents, thanks to the low surface roughness achieved after polishing [12]. This situation also explains the material’s high chemical resistance to acidic solutions such as cola and red wine. Papathanasiou et al. reported that the surface roughness of PEEK material did not show a statistically significant change at the end of the 30-day immersion period, regardless of the type of solution [16]. However, in our study, the increase in roughness observed in the GFP group suggests that, unlike pure PEEK, the fiber-matrix interface in some modified materials may be more sensitive to the liquid environment and surface stability may be compromised.

While PEEK materials provide an esthetic advantage by eliminating the gray reflection of metal frameworks, their optical properties may be insufficient to fully mimic natural tooth tissue. Ahmad et al. [22] stated that white or tooth-colored forms of PEEK are esthetically superior to metals, yet they still require veneering in regions with high esthetic expectations. The color changes observed in our study indicate that the esthetic success of PEEK material depends not only on the initial color but also on its long-term resistance to staining agents.

In the present laboratory investigation, several intraoral parameters could not be fully replicated, which constitutes a primary limitation. First, the surface properties of the materials were evaluated statically only after immersion, and no inter-analysis cleaning or dynamic brushing protocols were applied. In a clinical setting, daily patient hygiene procedures, such as mechanical toothbrushing and the use of chemical tablets or mouthwashes, can significantly alter surface characteristics; indeed, Porojan et al. reported that such procedures significantly reduce the Ra values of PEEK materials [23]. While mechanical brushing might partially compensate for stain accumulation, the simultaneous introduction of abrasive forces and dynamic thermal fluctuations from food and beverage intake can accelerate topographical alterations and matrix wear over time. Complex intraoral conditions—including the continuous presence of salivary pellicle, physiological pH changes, and parafunctional activities—exert a synergistic influence on the surface and optical stability of dental polymers. Because these multifactorial clinical dynamics and specific patient dietary frequencies were isolated in this static model, caution should be exercised when extrapolating these in vitro findings directly to long-term in vivo performance.

One of the variables in this study was the format of the raw materials, which required the GFP specimens to be prepared via precision cutting while the others were milled via CAD-CAM. Although a strict, standardized manual grinding and fine polishing protocol was applied to eliminate initial surface variations and establish a uniform baseline roughness across all groups, future studies utilizing identical manufacturing modalities for all evaluated polymers could provide further standardization regarding potential microstructure-level processing effects.

Furthermore, the omission of dynamic thermocycling and mechanical brushing protocols limits the immediate clinical extrapolation of these findings. While this static immersion successfully isolated the baseline chemical surface discoloration and pigment adsorption kinetics driven by distinct beverages over 30 days, the dynamic physical and thermal fluctuations of the intraoral environment must be carefully considered. Regarding these media properties, Papathanasiou et al. [16] maintained all solutions at a constant body temperature of 37 °C. In contrast, our study utilized a more clinically realistic approach where coffee and tea were consumed hot, while cola and wine were served cold. This temperature variation is critical, as elevated temperatures are known to accelerate pigment adsorption and alter surface tension within the polymer matrix [41]. Therefore, while our protocol closely mirrors real-life dietary habits, the absence of continuous thermal cycling software during the 30-day immersion remains a limitation that should be addressed alongside dynamic physical abrasion in future multi-factorial simulation designs.

## 5. Conclusions

Concerning the results of the present study and according to its limitations, it was concluded that:

- The type of modified PEEK material and the type of beverage solution have a significant effect on color stability and surface roughness.
- Selecting clinically optimized PEEK variants, such as TP and CR, can effectively extend the functional lifespan of fixed and implant-supported prostheses.
- GFP exhibited the highest susceptibility regarding both color instability and increased surface roughness.
- More studies are required to comprehensively evaluate the long-term clinical and esthetic performance of such materials.

Due to the limited number of studies in the literature on this subject, especially regarding modified high-performance polymers, this study fills an important gap. To obtain more precise and comparable results in future investigations, further long-term in vitro and dynamic clinical research that closely simulates the complex oral environment is essential.

## Figures and Tables

**Table 1:** Formation of groups.

Materials	Solutions	n	Abbreviation	Total
%100 PEEK (P) (PEEK-OPTIMA™, Juvora Ltd., Thornton-Cleveleys, UK)	Water	7	P-DW	35
	Coffee	7	P-CF	
	Tea	7	P-T	
	Cola	7	P-CC	
	Red wine	7	P-RW	
%20 TiO_2_ PEEK (TP) (KERA^®^starPEEK, Wörth am Main, Germany)	Water	7	TP-DW	35
	Coffee	7	TP-CF	
	Tea	7	TP-T	
	Cola	7	TP-CC	
	Red wine	7	TP-RW	
%20 Nano Ceramic PEEK (CR) (BioHPP, Bredent GmbH, Senden, Germany)	Water	7	CR-DW	35
	Coffee	7	CR-CF	
	Tea	7	CR-T	
	Cola	7	CR-CC	
	Red wine	7	CR-RW	
%30 Glass Fiber PEEK (GFP) (TECAPEEK GF3O Naturel, Ensinger, Nufringen, Germany)	Water	7	GFP-DW	35
	Coffee	7	GFP-CF	
	Tea	7	GFP-T	
	Cola	7	GFP-CC	
	Red wine	7	GFP-RW	
Total				140

**Table 2:** Statistical analysis table of percentage change in ΔE and Ra.

Materials	Solutions	n	ΔE	Percentage Change in Ra
P	Water	7	1.566 ± 0.271	12.863 ± 2.343
	Coffee	7	4.144 ± 0.212	37.557 ± 3.216
	Tea	7	3.706 ± 0.200	30.507 ± 3.895
	Cola	7	3.456 ± 0.060	21.263 ± 1.471
	Red wine	7	3.254 ± 0.087	25.501 ± 2.404
TP	Water	7	1.234 ± 0.089	4.153 ± 4.823
	Coffee	7	3.594 ± 0.347	18.016 ± 8.272
	Tea	7	3.259 ± 0.229	19.516 ± 11.698
	Cola	7	2.519 ± 0.075	20.266 ± 11.033
	Red wine	7	3.119 ± 0.316	22.404 ± 12.022
GFP	Water	7	1.573 ± 0.058	44.517 ± 9.543
	Coffee	7	5.856 ± 0.435	99.440 ± 8.873
	Tea	7	14.52 ± 0.407	91.881 ± 25.868
	Cola	7	3.584 ± 0.479	20.560 ± 7.371
	Red wine	7	4.363 ± 0.349	63.326 ± 2.070
CR	Water	7	1.243 ± 0.483	1.179 ± 0.935
	Coffee	7	4.113 ± 0.579	14.387 ± 3.335
	Tea	7	3.673 ± 0.385	19.016 ± 1.457
	Cola	7	3.124 ± 0.620	16.347 ± 3.029
	Red wine	7	3.254 ± 0.324	12.653 ± 1.080
Materials				
P		35	3.225 ± 0.911 ^b^	25.538 ± 8.862 ^b^
TP		35	2.745 ± 0.873 ^c^	16.871 ± 11.446 ^c^
GFP		35	5.979 ± 4.568 ^a^	63.945 ± 32.307 ^a^
CR		35	3.081 ± 1.098 ^b^	12.716 ± 6.571 ^c^
Solutions				
Water		35	1.404 ± 0.315 ^E^	15.678 ± 18.260 ^C^
Coffee		35	4.427 ± 0.953 ^B^	42.350 ± 35.280 ^A^
Tea		35	6.289 ± 4.852 ^A^	40.230 ± 33.572 ^A^
Cola		35	3.171 ± 0.561 ^D^	19.609 ± 6.742 ^C^
Red wine		35	3.498 ± 0.580 ^C^	30.971 ± 20.489 ^B^
ANOVA			*p* value	
Material			<0.0001	<0.0001
Solution			<0.0001	<0.0001
Material x Solution			<0.0001	<0.0001

a–c: The difference between materials carrying different letters in the same row is statistically significant (*p* < 0.05). A–E: The difference between beverages carrying different letters in the same row is statistically significant (*p* < 0.05).

## Data Availability

The original contributions presented in this study are included in the article. Further inquiries can be directed to the corresponding author.
